# In Situ Laser-Induced Fabrication of a Ruthenium-Based Microelectrode for Non-Enzymatic Dopamine Sensing

**DOI:** 10.3390/ma13235385

**Published:** 2020-11-27

**Authors:** Maxim S. Panov, Anastasiia E. Grishankina, Daniil D. Stupin, Alexey I. Lihachev, Vladimir N. Mironov, Daniil M. Strashkov, Evgeniia M. Khairullina, Ilya I. Tumkin, Mikhail N. Ryazantsev

**Affiliations:** 1 Saint Petersburg State University, 7/9 Universitetskaya nab., 199034 St. Petersburg, Russia; m.s.panov@spbu.ru (M.S.P.); n.grshnk@gmail.com (A.E.G.); vova_mironov_97@mail.ru (V.N.M.); e.khayrullina@spbu.ru (E.M.K.); 2 Nanotechnology Research and Education Centre RAS, Saint Petersburg Academic University, 8/3 Khlopina Street, 194021 St. Petersburg, Russia; stu87@yandex.ru (D.D.S.); danielstr@mail.ru (D.M.S.); 3 Ioffe Institute, 26 Politekhnicheskaya, 194021 St. Petersburg, Russia; lihachev@mail.ioffe.ru

**Keywords:** laser-induced metal deposition, ruthenium, dopamine, non-enzymatic sensors

## Abstract

In this paper, we propose a fast and simple approach for the fabrication of the electrocatalytically active ruthenium-containing microstructures using a laser-induced metal deposition technique. The results of scanning electron microscopy and electrical impedance spectroscopy (EIS) demonstrate that the fabricated ruthenium-based microelectrode had a highly developed surface composed of 10 μm pores and 10 nm zigzag cracks. The fabricated material exhibited excellent electrochemical properties toward non-enzymatic dopamine sensing, including high sensitivity (858.5 and 509.1 μA mM^−1^ cm^−2^), a low detection limit (0.13 and 0.15 μM), as well as good selectivity and stability.

## 1. Introduction

The fabrication of new materials for the detection of various biologically important analytes is of great interest in medical diagnostics, science, and industry [1,2]. Dopamine is one such analyte, along with glucose, hydrogen peroxide, amino acids, and many others [3]. Dopamine is the key catecholamine neurotransmitter released by the brain and it plays a crucial role in the functioning of several biological systems, including the central nervous system. An abnormal level of this biogenic amine in human blood is implicated in the development of a number of neurological diseases. Indeed, insufficient dopamine in the brain may lead to schizophrenia, Alzheimer’s disease, and Parkinson’s disease, whereas high levels of dopamine result in Huntington’s disease [4]. Many methods can be used to determine the concentration of this and other disease markers in physiological fluids and model solutions, for example, high-performance liquid chromatography–mass spectroscopy (HPLC-MS) [5], fluorometry [6], colorimetry [7], and voltamperometry [8]. Despite their capability to detect low concentrations of an analyte, almost all of these techniques exhibit severe drawbacks: they are expensive, time consuming, and can complicate experimental procedures. In turn, electrochemical methods are thought to be among the most effective methods of detecting low concentrations of many disease markers, including dopamine, primarily due to their high sensitivity and fast response [3,9,10]. As a rule, the electrochemically based techniques operate either in an enzymatic or in a non-enzymatic regimen [11]. Despite decent specificity, enzymatic sensing of dopamine has a number of limitations, such as low accuracy of detection, poor reproducibility and low stability as a result of enzyme decomposition (typically, tyrosinase) [12], and being subject to influence of the environment (pH and temperature) [11]. In contrast, in the enzyme-free mode, direct detection of dopamine is ensured by catalysis of ox-red reactions of this analyte occurring on the surface of an electrode [13]. In order to achieve the best effect in determining dopamine levels, an electrode must have a highly developed surface area, ultimately allowing for a significant increase in sensitivity and stability, as well as reduced values of detection potentials. However, the problem of low selectivity associated with electrochemical methods remains unsolved [14].

Numerous approaches can be used to manufacture materials with surface areas that demonstrate a high degree of porosity. Among them are inkjet printing [15], direct laser writing (DLW) [16], selective laser sintering (SLS) [17], screen printing [18], and chemical vapor deposition (CVD) [19]. Despite their many merits, some of these methods have substantial shortcomings, such as expensive reagents and equipment [15,18,19], as well as insufficient adhesion properties of the produced structures [17] and low deposition rates [19]. On the contrary, we propose a simple and inexpensive method that has practically no such disadvantages. This method deals with laser-induced deposition of a metal from a solution on the surface of various dielectric substrates (LCLD) [20,21]. In general, the main feature of LCLD is that the reduction reaction of a metal and its subsequent deposition on the surface of glass, glass ceramics or other dielectric materials occurs in a local volume of a solution within the focus of a laser beam. Accordingly, it is possible to synthesize metallic and bimetallic microstructures of different phase composition having a highly developed surface area and, as a result, exhibiting high electrocatalytic activity toward various analytes. Previously, we were able to fabricate sensor platforms based on copper [22], nickel [23], gold [24], platinum [25], iridium [25], molybdenum [26], silver [27], and cobalt [28], which are appropriate for glucose, hydrogen peroxide, and alanine enzymeless sensing. In the current study, we manufactured a ruthenium-based microelectrode to detect dopamine concentration. Materials containing ruthenium are widely known and are used as enzyme-free sensors [29,30,31,32,33,34,35,36]. For example, porous ruthenium oxide (RuO_2_) is used to catalyze glucose and hydrogen peroxide, as well as to measure pH [30]. This usefulness is due to the fact that RuO_2_ exhibits high sensitivity, good electrocatalytic activity, outstanding thermal stability, and high corrosion resistance. In addition, it was recently shown that ruthenium disulfide (RuS_2_) demonstrates high sensitivity with respect to dopamine detection due to its great stability, electronic configuration nature, availability of catalytic active sites, and superb electrochemical redox characteristics [36]. Thus, as a main part of this work, we developed a sensor platform based on ruthenium microstructures with good selectivity, decent stability, and high sensitivity to the non-enzymatic determination of dopamine.

## 2. Materials and Methods

### 2.1. Materials

The laser-induced deposition of ruthenium-based microstructures on the surface of glass was performed using a solution containing 3 mM of triruthenium dodecacarbonyl (Ru_3_(CO)_12_) in *N*,*N*-dimethylformamide (DMF). These reagents were analytically graded and were purchased from Sigma Aldrich (St. Louis, MO, USA) for further usage without any additional purification.

### 2.2. Synthesis of Ru-Based Microelectrode

**Figure 1:**
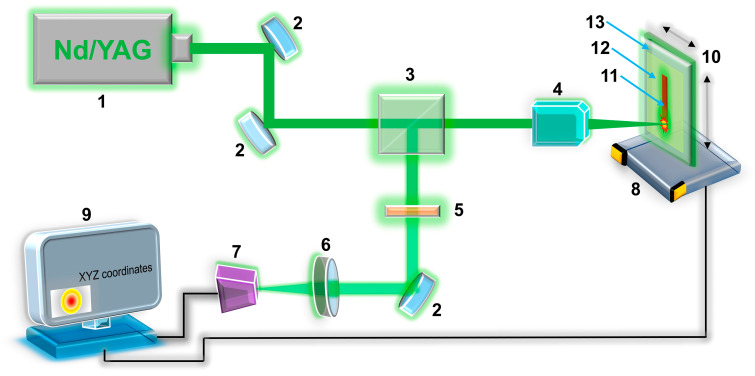
The schematic illustration of the experimental setup for the fabrication of the Ru-based microelectrode: (1) diode-pumped continuous-wave solid-state Nd:YAG 532 nm laser; (2) aluminum mirror; (3) optical separation cube; (4) microscope objective; (5) ND filter; (6) lens; (7) web-camera; (8) computer-controlled XYZ motorized stage; (9) personal computer (PC); (10) experimental cell; (11) fabricated Ru-microelectrode; (12) 3 mM Ru_3_(CO)_12_ in DMF; (13) glass substrate.

The principal scheme of the synthesis setup is shown in Figure 1. A diode-pumped continuous-wave solid-state Nd:YAG laser (Changchun, China) operating at 532 nm was used as a light source for thermally induced reduction and deposition of Ru microstructures. The laser output traveled through two aluminum mirrors and an optical separation cube, and was then focused on the sample (solution) using a standard microscope objective with a focal length of 15 mm. The solution containing a ruthenium(VI) carbonyl complex was placed in a special experimental cell, which was moved by a computer-controlled XYZ-motorized platform. Further, part of the laser output was reflected back by the cell and was redirected by an optical separation cube toward a web-camera for in situ monitoring of the laser metal deposition process. Here, the neutral-density (ND, fractional transmittance 25%) filter was inserted into an optical path in order to prevent optical damage to the camera by an excess of the 532 nm light. Finally, ruthenium microstructures were produced by scanning a laser beam focused on the solution–glass interface along the vertical direction of the cell movement. Because of such laser writing, we were able to synthesize a ruthenium microelectrode with a length of ~10 mm and a width of ~100 μm at a laser power of 1400 mW and a scanning speed of 7.5 μm s^−1^.

### 2.3. Morphology and Phase Identification of Ru-Based Microelectrode

The morphology of the fabricated Ru-based microelectrode was investigated using a scanning electron microscope JSM-7001F (SEM, JEOL, Japan) coupled with an energy-dispersive analyzer INCA PentaFETx (Oxford Instruments, UK) to characterize its atomic composition.

The X-ray diffraction analysis (XRD) for phase identification of the synthesized ruthenium material was performed on a Bruker D2 Phaser diffractometer equipped with a LynxEye detector (Bruker-AXS, Karlsruhe, Germany) using CuKα (0.1542 nm) radiation in the 2θ angle range of 0°–100°.

### 2.4. Impedance Measurements

**Figure 2:**
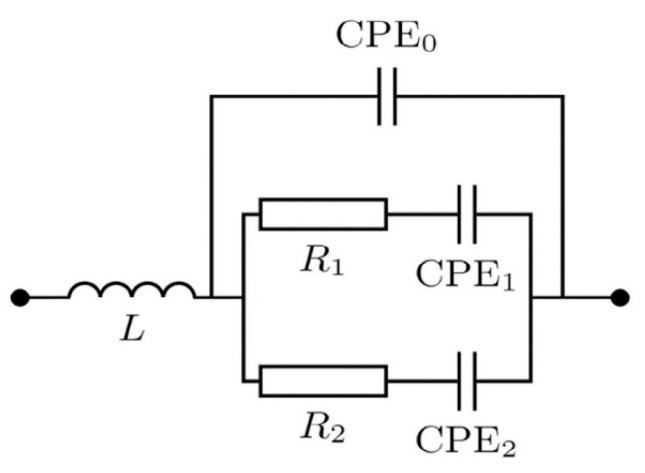
The equivalent scheme for describing the ruthenium microelectrode. Here, *L* = 3 mH is the parasitic inductance caused by the finite-time response of the ammeter.

For obtaining impedance spectra using high-speed and high-resolution EIS methods [37]—AF-EIS [38] and Fourier-EIS [39]—a homemade setup was used [38]. The measurements were provided with 15 mV sweep-shape excitation voltage in the frequency range of 100 Hz to 40 kHz with a 2 Hz resolution. To create the electrochemical cell, a ruthenium-based microelectrode and a Pt reference electrode with a large surface area were embedded into glass containing 0.9% NaCl solution (Biolot, St. Petersburg, Russia). The impedance spectra approximation by the complex non-linear least squares (CNLS) method [40] was made in the NELM package for MATLAB [38] (available upon request). Figure 2 demonstrates the scheme used for the CNLS spectra analysis. Here, CPE is constant phase element [41], the impedance of which equals:

(1) $$Z=\frac{1}{W{\left(i\omega \right)}^{\alpha }}$$

where *α* is the non-ideality parameter and *W* is the pseudo-capacitance with dimension *S s^α^*. Typically, CPE elements describe non-ideal capacitors. In particular, *α* ≈ 0.5 can refer to the interface between electrolyte and electrode with the developed (porous) surface [42,43,44]. To account for the delay between excitation voltage and current response measurements by ADC (analog-to-digital converter), the parameter *Δt* was introduced in the model as follows:

(2) $$Y_{m}=Y_{s}\times e^{i\omega \Delta t}$$

where *Y_m_* is the model, which was used for CNLS approximation, *Y_s_* is the admittance (Figure 2), and *ω* is the angular frequency. The measurements were repeated 10 times in order to obtain statistics.

### 2.5. Electrochemical Studies

The electrochemical properties of the fabricated Ru-based microstructures were studied using voltammetric methods. All measurements were carried out on an Elins P30I potentiostat (Electrochemical Instruments Ltd., Chernogolovka, Russia) at an ambient temperature in a standard three-electrode cell, in which platinum wire, an Ag/AgCl electrode, and a ruthenium microelectrode were used as counter, reference, and working electrodes, respectively. Cyclic voltammetric studies were run at a scan rate of 50 mV s^−1^ between −0.9 and 0.9 V vs. Ag/AgCl. Amperometric responses were recorded by adding dopamine of various concentrations to the background solution (0.1 M NaOH) with simultaneous stirring. D-glucose, ascorbic acid, urea, and hydrogen peroxide were used as interfering components when determining the selectivity of the Ru-based microelectrode toward dopamine.

## 3. Results and Discussion

The conductive ruthenium microstructures were fabricated by means of LCLD after optimization of the experimental conditions, i.e., at a laser power of 1400 mW, at a scanning speed of 7.5 μm s^−1^, and using 3 mM Ru_3_(CO)_12_ in DMF. It should be noted that we were able to produce metal structures at a significantly higher scanning speed compared with other sensor-active materials previously synthesized using LCLD (3 times faster, 0.75 vs. 0.25 μm s^−1^). We did not expect photochemical reactions to contribute to the deposition process because the DMF solution of the ruthenium carbonyl complex used in this work was transparent to the 532 nm laser light.

**Figure 3:**
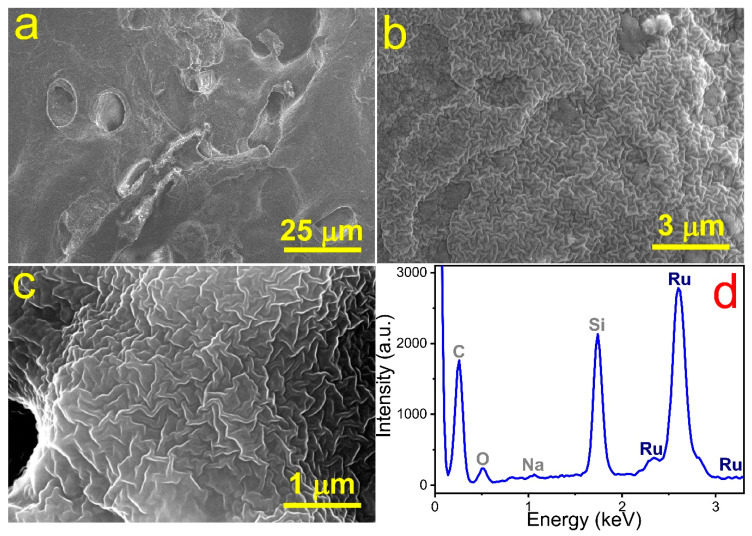
The SEM images (**a**–**c**) and EDX spectrum (**d**) of the fabricated Ru-based microelectrode. The morphology analysis revealed that the electrode surface has large pores and small zigzag cracks. According to the elemental analysis, Ru-based microstructures mostly consist of ruthenium with weight percentage (wt.%) of 29.

**Figure 4:**
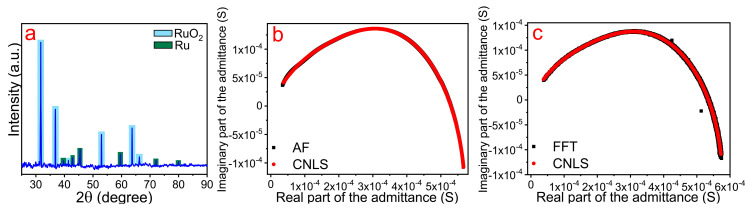
(**a**) The XRD pattern of the Ru-based microstructures deposited on glass; (**b**) The admittance spectrum of the Ru electrode obtained using the AF-EIS method; (**c**) The admittance spectrum of the Ru electrode obtained using the Fourier-EIS method. For both methods, the black squares correspond to the experimental value, whereas the red circles refer to the CNLS approximation. Both the experimental techniques provide low-noise data, which can be perfectly fitted using the scheme illustrated in Figure 2.

The results of the Ru-based microelectrode surface analysis using scanning electron microscopy (SEM) and energy-dispersive X-ray spectroscopy (EDX) are presented in Figure 3. Here, one can see that the electrode had a non-flat, complex surface with two levels of development; specifically, it had large-scale 10 μm pores (Figure 3a,b) and small-scale 10 nm surface irregularity (Figure 3c). According to EDX data, the manufactured electrode was mainly composed of ruthenium and partially of oxygen (Figure 3d). The peaks at 0.26, 1.06, and 1.74 keV corresponded to carbon, sodium, and silicon, respectively, the presence of which can be attributed to the substrate material (glass). These findings were supported by the X-ray diffraction analysis. The observed XRD pattern (Figure 4a) demonstrated that the fabricated ruthenium microstructures contained both metallic and oxide (RuO_2_) phases. In turn, the presence of ruthenium dioxide may possibly explain the relatively high values of the electrical resistance of the microelectrode (~1.2 kΩ) and its semiconductor nature. However, more detailed studies are required for a better understanding.

We evaluated the porosity of the resulting ruthenium electrode using impedance spectroscopy as the most important criterion for its further application as an enzyme-free sensor. The obtained spectra and the approximated elements of the equivalent scheme are shown in Figure 4b,c and Table 1 and Table 2, respectively. From Figure 4b,c, it is clear that the three-branch scheme in Figure 2 (CPE_0_, *R*_1_-CPE_1_, *R*_2_-CPE_2_) gave a perfectly fitting result for both AF-EIS and Fourier-EIS. We considered every *R*-CPE branch in the scheme illustrated in Figure 2. First, we observed that the CPE_0_ branch has *α*_0_ ≈ 1. Moreover, the value of the *W*_0_ was close to those of the capacity of the wires used as the contacts with the sample. Thus, the CPE_0_ branch corresponded to the parasitic capacity leakage in the wires. Second, the *α*-values of the *R*_1_-CPE_1_ and *R*_2_-CPE_2_ branches were significantly lower than unity. This observation indicated that the surface of the ruthenium electrode consisted of two phases with different degrees of porosity—something confirmed by the SEM images in Figure 3a–c, in which one can see two types of structures on the surface of the Ru electrode: 10 μm scale pores and 10 nm scale zigzag cracks. Therefore, the equivalent scheme in Figure 2 (except parasitic inductance and the CPE_0_ branch) could be directly associated with the Ru electrode’s surface morphology. In another words, the electrical properties of the electrode material were in agreement with the properties of its surface morphology. Indeed, *α*_1_ of the *R*_1_-CPE_1_ branch corresponded to the more developed part of the electrode surface, whereas the value of *α*_2_ obtained from *R*_2_-CPE_2_ was associated with those areas that have a lower degree of surface development. Furthermore, both the *R*_1_-CPE_1_ and *R*_2_-CPE_2_ branches provided an equal contribution to admittance and thus took into account that these two branches were important for Ru-based microelectrode characterization.

**Figure 5:**
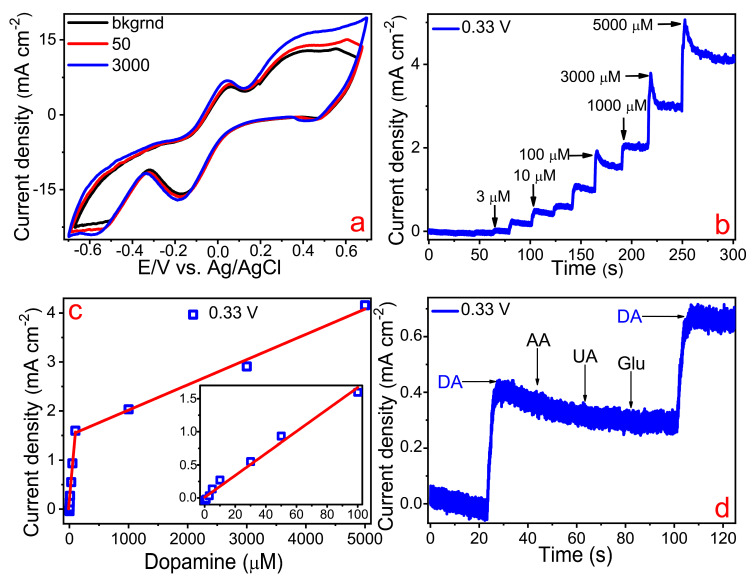
(**a**) CVs of the Ru electrode recorded at two concentrations of dopamine; (**b**) Amperogram of the Ru electrode recorded in the presence of different concentrations of dopamine at the potential of 0.33 V; (**c**) Linear dependence of the measured amperometric current on the dopamine concentrations; (**d**) The response of the amperometric current to the consecutive addition of 10 μM dopamine (DA), 3 μM ascorbic acid (AA), 3 μM uric acid (UA), and 3 μM D-glucose (Glu) in a background solution of 0.1 M NaOH.

We studied the electrochemical properties of the synthesized Ru electrode. Figure 5a shows the cyclic voltammograms of the ruthenium microstructures in dopamine solutions of various concentrations. A typical cyclic voltammogram (CV) has pronounced anode and cathode peaks of dopamine. Two regions of anodic oxidation can be distinguished as follows: the first range lay between −0.14 and 0.12 V, whereas the second interval of oxidation potentials was between 0.13 and 0.52 V. These regions can possibly be attributed to two electrocatalytic oxidation processes: Ru^2+^/Ru^3+^ and Ru^0^/Ru^3+^, respectively. Using the direct amperometry method, we obtained such important electrochemical parameters as the limit of detection and the sensitivity of the fabricated microelectrode to enzyme-free dopamine sensing. Figure 5b illustrates a typical amperometric signal showing how the successive additions of dopamine of different concentrations to a background solution at a potential of 0.33 V changed the Faraday current. It is clear that the Faraday current increased as the dopamine concentration increased; in turn, linear intervals of such change for the Ru electrode lay in 1–100 and 100–5000 µM. The detection limits (LOD) of dopamine for the ruthenium-based microelectrode were calculated as *LOD = 3S/b*, where *S* is the standard deviation from linearity, whereas *b* is the slope of the calibration curve (the linear ranges are shown in Figure 5c). Thus, the calculated LOD values for these two intervals were 0.13 and 0.15 µM, respectively. The maximum calculated sensitivities attributed to these linear ranges were 858.5 and 509.1 µA mM^−1^ cm^−2^, respectively. It is known that the CV area and, consequently, the sensitivity are directly associated with the degree of development of the electrode surface. Therefore, the low detection limit and high sensitivity revealed by the ruthenium electrode can be explained by the high porosity of this material. The recorded electrocatalytic parameters of the Ru electrode were compared with several electrode materials that were used for dopamine enzymeless sensing [36,45,46,47,48,49] (Table 3).

We also tested the selectivity of the Ru-based microelectrode in the presence of a number of interfering substances, such as ascorbic acid (AA), urea (UA), and D-glucose (Glu). Figure 5c illustrates that the most pronounced change in the Faraday current was observed by the addition of dopamine to the background solution as opposed to other tested analytes. This means that the fabricated electrode may have quite decent selectivity regarding dopamine detection both in the model solutions and in human blood.

**Figure 6:**
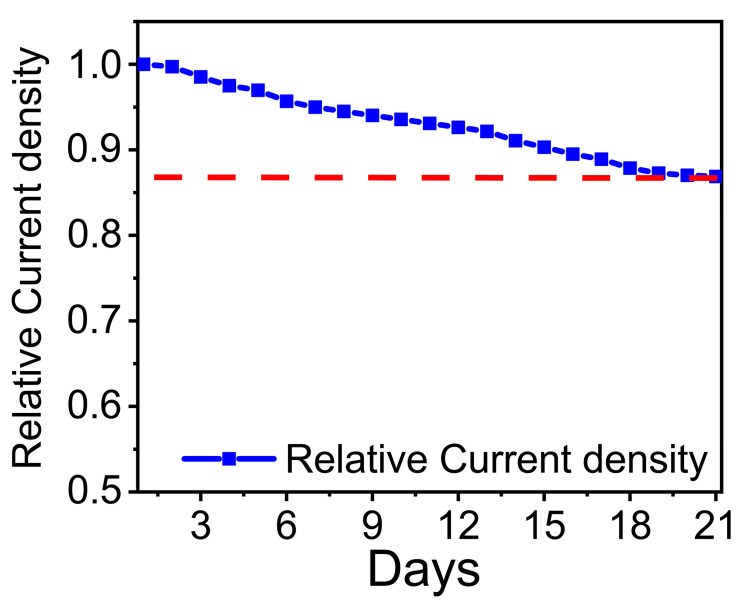
Long-term stability of the Ru-based sensor for enzyme-free dopamine detection observed during three weeks.

The long-term stability of the Ru-based microelectrode stored under ambient conditions was studied by measuring the relative current density I_x_/I_1_, where I_x_ and I_1_ are amperometric responses of the developed electrode toward the addition of 10 µM dopamine recorded on the first day and each following day during the subsequent three weeks. It was observed that the relative current density remained above 86% of its initial value after three weeks of testing revealing an acceptable level of Ru-based microelectrode stability (Figure 6).

## 4. Conclusions

Laser-induced metal deposition was used to fabricate ruthenium microstructures that had good electrocatalytic properties with respect to dopamine detection. These structures were deposited on a glass surface within the focus of a laser beam at a wavelength of 532 nm and with a sufficiently high scanning speed. Morphological studies showed that the resulting ruthenium deposits had a highly developed surface with hierarchical structures, which were proved by the presence of two sets of pores: 10 μm large pores and small 10 nm zigzag cracks. The elemental and phase analysis demonstrated that the fabricated material had not only a metallic phase, but also contained ruthenium dioxide, which can serve as evidence of high values of electrical resistance. In turn, the high active surface area of the Ru-based microstructure explained its high electrocatalytic activity toward the enzyme-free determination of dopamine, which was confirmed by voltammetric studies. It was found that Ru-based microelectrode had two linearity ranges (1–100 and 100–5000 μM). Within these ranges, the fabricated electrode revealed sufficiently low values of detection limits (0.13 and 0.15 μM) and high values of sensitivity (858.5 and 509.1 μA mM^−1^ cm^−2^). In addition, the Ru-based microelectrode exhibited good stability and great selectivity in the presence of a number of interfering analytes, including ascorbic acid. Thus, the manufactured material can be considered as sufficiently promising for the development and design of sensor platforms for non-enzymatic sensing of dopamine and possibly for other important disease markers.

## Figures and Tables

**Table 1:** Approximation results for the admittance spectrum obtained using the AF-EIS model.

**Parameter**	*R*_1_, Ω	*W*_1_, S s*^α^*^1^	*α*_1_
**Value**	4.4 × 10^3^	1.09 × 10^−6^	0.604
**Relative Error, %**	7	5	1
**Parameter**	-	*W*_0_, S s*^α^*^0^	*α*_0_
**Value**	-	4 × 10^−10^	1.02
**Relative Error, %**	-	50	4
**Parameter**	*R*_2_, Ω	*W*_2_, S s*^α^*^2^	*α*_2_
**Value**	2.7 × 10^3^	1.1 × 10^−7^	0.70
**Relative Error, %**	7	27	3

**Table 2:** Approximation results for the admittance spectrum obtained using the Fourier-EIS model.

**Parameter**	*R*_1_, Ω	*W*_1_, S s*^α^*^1^	*α*_1_
**Value**	3.8 × 10^3^	1.27 × 10^−6^	0.590
**Relative Error, %**	6	6	1
**Parameter**	-	*W*_0_, S s*^α^*^0^	*α*_0_
**Value**	-	6 × 10^−10^	0.99
**Relative Error, %**	-	50	5
**Parameter**	*R*_2_, Ω	*W*_2_, S s*^α^*^2^	*α*_2_
**Value**	3.1 × 10^3^	7 × 10^−8^	0.73
**Relative Error, %**	10	29	3

**Table 3:** Comparison of the electrochemical parameters of some electrode materials used for enzyme-free dopamine detection.

**Material of Electrode**	**Linear Range (μM)**	**LOD (μM)**	**Sensitivity (**μA mM^−1^ cm^−2^**)**	**References**
Ru	1–100 and 100–5000	0.13 and 0.15	858.5 and 509.1	This work
PPy/graphene composite	100–1000	2.3	363	[45]
Au@ZIF-8 nanocomposite	0.1–50	0.01	6.452	[46]
RuS2 NPs	10–80	0.0738	1800	[36]
PtNi-MoS2	0.5–250	0.1	502	[47]
Nf-Ag@HCS(hollow carbon spheres)/GCE	3–2000	0.6	757.4	[48]
Pd-NC/rGO	20–220	7.02	0.943	[49]
